# Role of hydroxymethylglutharyl-coenzyme A reductase in the induction of stem-like states in breast cancer

**DOI:** 10.1007/s00432-024-05607-7

**Published:** 2024-02-28

**Authors:** María Paula Marks, Carla Alejandra Giménez, Luciana Isaja, Mariana Belén Vera, Francisco Raúl Borzone, Federico Pereyra-Bonnet, Leonardo Romorini, Guillermo Agustín Videla-Richardson, Norma Alejandra Chasseing, Juan Carlos Calvo, Luciano Vellón

**Affiliations:** 1 Laboratorio de Células Madre/Stem Cells Lab (IBYME), Consejo Nacional de Investigaciones Científicas y Técnicas (CONICET), Instituto de Biología y Medicina Experimental, Vuelta de Obligado 2490, CP 1428 Ciudad Autónoma de Buenos Aires, Argentina; 2 Instituto de Ciencias Básicas y Medicina Experimental, Instituto Universitario del Hospital Italiano, Potosí 4265, C1199ACL Buenos Aires, Argentina; 3 Laboratorio de Investigación Aplicada a Las Neurociencias (LIAN), Fundación Para La Lucha Contra Las Enfermedades Neurológicas de La Infancia (FLENI), Ruta 9, Km 53, B1625 Buenos Aires, Escobar Argentina; 4 Laboratorio de Inmunohematología, (IBYME), Consejo Nacional de Investigaciones Científicas y Técnicas (CONICET), Instituto de Biología y Medicina Experimental, Vuelta de Obligado 2490, CP 1428 Ciudad Autónoma de Buenos Aires, Argentina; 5 Present Address: CASPR Biotech, Buenos Aires, Argentina; 6 Present Address: CASPR Biotech, San Francisco, USA

**Keywords:** Breast cancer, Cancer stem cells, HMGCR, Metabolic reprogramming

## Abstract

**Purpose:**

De novo synthesis of cholesterol and its rate-limiting enzyme, 3-hydroxy-3-methylglutharyl-coenzyme A reductase (HMGCR), is deregulated in tumors and critical for tumor cell survival and proliferation. However, the role of HMGCR in the induction and maintenance of stem-like states in tumors remains unclear.

**Methods:**

A compiled public database from breast cancer (BC) patients was analyzed with the web application SurvExpress. Cell Miner was used for the analysis of HMGCR expression and statin sensitivity of the NCI-60 cell lines panel. A CRISPRon system was used to induce HMGCR overexpression in the luminal BC cell line MCF-7 and a lentiviral pLM-OSKM system for the reprogramming of MCF-7 cells. Comparisons were performed by two-tailed unpaired t-test for two groups and one- or two-way ANOVA.

**Results:**

Data from BC patients showed that high expression of several members of the cholesterol synthesis pathway were associated with lower recurrence-free survival, particularly in hormone-receptor-positive BC. In silico and in vitro analysis showed that HMGCR is expressed in several BC cancer cell lines, which exhibit a subtype-dependent response to statins in silico and in vitro. A stem-like phenotype was demonstrated upon HMGCR expression in MCF-7 cells, characterized by expression of the pluripotency markers NANOG, SOX2, increased CD44 +/CD24low/ −, CD133 + populations, and increased mammosphere formation ability. Pluripotent and cancer stem cell lines showed high expression of HMGCR, whereas cell reprogramming of MCF-7 cells did not increase HMGCR expression.

**Conclusion:**

HMGCR induces a stem-like phenotype in BC cells of epithelial nature, thus affecting tumor initiation, progression and statin sensitivity.

**Supplementary Information:**

The online version contains supplementary material available at 10.1007/s00432-024-05607-7.

## Introduction

Alterations in lipid metabolism have gained attention as main players in many aspects of cancer growth, including cancer stem cells (CSCs) states. Indeed, hypercholesterolemia can lead to cancer by several means, including cholesterol derived-oncometabolites (Silvente-Poirot et al. 2018), or an increased but regulated uptake of cholesterol in the form of low-density lipoprotein (LDL)-cholesterol (Scully et al. 2022). In addition, enhanced endogenous biosynthesis of cholesterol and isoprenoids appears to play a role in cancer initiation and progression (Göbel et al. 2022), since many enzymes in the mevalonic acid (MVA) pathway such as 3-hydroxy-3methylglutaryl-coenzyme A reductase (HMGCR), farnesyl diphosphate synthase, geranylgeranyl pyrophosphate synthase, squalene synthase and squalene epoxidase are overexpressed and overactivated in melanoma (Kuzu et al. 2016), glioblastoma (Abate et al. 2017), lung (Wang et al. 2018), colon (Gao et al. 2021), prostate (Todenhöfer et al. 2013), ovarian (de Wolf et al. 2017) and breast cancer (BC) (Brown et al. 2016). Moreover, in the seminal work by Clendening et al. (2010), it was demonstrated that overexpression of the rate-limiting enzyme of the MVA pathway and the target of the lipid-lowering drugs statins, HMGCR, promoted malignant transformation in BC. A more recent study in patient-derived xenograft tumors (PDXs) from ER-negative BC confirmed that the cholesterol biosynthesis pathway was critical to breast CSCs propagation and a potential therapeutic target (Ehmsen et al. 2019).

The CSCs hypothesis postulates that tumors are hierarchically organized, with a small fraction of stem cells at the apex of the hierarchy, and rapidly proliferating and differentiated, post-mitotic cells constituting the bulk of the tumor (Clevers 2011; Azizidoost et al. 2023). Particularly, BC stem cells (BCSCs) are a small population of BC cells that share many traits with normal mammary stem cells, play a critical role in the metastasis of BC to other organs in the body, have the ability to self-renew, differentiate to give rise to phenotypically diverse cells, and are resistant to conventional anti-cancer treatments, thus being implicated in disease recurrence and metastasis (Song and Farzanehl 2021). Currently, the view of the CSCs model incorporates the concept of cellular plasticity (potential for cellular reprogramming), suggesting “stemness” would be a cellular state capable of being switched on or off in response to cell-intrinsic and/or microenvironmental cues (Corominas-Faja et al. 2013; Vazquez-Martin et al. 2013).

Being a target for HMGCR inhibitors, the MVA pathway may exert interesting therapeutic potential for treatment and prevention of BC, since statins can affect a wide range of molecular processes such as inflammation, cell migration, proliferation, apoptosis, angiogenesis and stemness by means of cholesterol-mediated and non-mediated pathways (Zaky et al 2023). In this regard, it has been shown that treatment with Simvastatin (SIM) decreased the number of CSCs and formation of mammospheres from drug-resistant cells and from PDX tumors (Gopalan et al. 2013; Ehmsen et al. 2019) and decreased the expression of CSC markers in an in vivo model (Rennó et al. 2015). Moreover, Lovastatin (LOVA) was identified as a CSC-targeting drug (Vásquez-Bochm et al. 2019). These results imply that the MVA pathway is implicated in the enrichment of CSCs, and that anti-cholesterol therapies may be able to eliminate the stem compartment of the tumor. Therefore, in the present work, we aimed to elucidate the contribution of HMGCR to the induction and maintenance of CSC-states and statin response in BC.

## Materials and methods

### Public databases

A compiled database (Breast Cancer Metabase 10 cohorts 22 K genes; *n* = 1901) was analyzed with the web application SurvExpress from the Tecnológico de Monterrey, México (Aguirre-Gamboa et al. 2013), (Cuéllar et al. 2015). Expression of the following MVA pathway enzymes was assessed: Acetyl-CoA acetyltransferase (ACAT2), hydroxymethylglutharyl Acetyl-CoA synthase 1 (HMGCS1), HMGCR, mevalonate kinase (MVK), phosphomevalonate kinase (PMVK), mevalonate pyrophosphate decarboxylase (MVD), isopentenyl diphosphate Delta isomerase 1 (IDI1), FDPS, GGPS1, FDFT1, SQLE, Lanosterol Synthase (LSS), 7-dehydrocholesterol Reductase (DHCR7) and Delta(24)-sterol Reductase (DHCR24). Patients were dichotomized on the basis of the corresponding mRNA abundance and Kaplan Meier (KM) curves were plotted with differences in outcome calculated using a Cox proportional hazard model. The web application Cell Miner from the National Cancer Institute (NCI 2020) was used to determine the levels of HMGCR and the relative drug activity of SIM, LOVA, Atorvastatin (ATOR), Fluvastatin (FLUV) and Mevastatin (MEVA) across 60 cancer cell lines (NCI-60 panel). Results are expressed as Z-scores as previously described (Reinhold et al. 2012).

### Plasmids, cell culture and transfections

The sgRNA expression plasmid (pSPgRNA) and the plasmid encoding dCas9-VP160 (pAC94-pmax-dCas9-VP160-2A-puro) were developed by others (Perez-Pinera et al. 2013; Cheng et al. 2013) and obtained from Addgene (plasmids #47108 and #48226). The system was generated as previously described (Giménez et al. 2016). For more details, see Supplementary Fig.S1. For cell reprogramming experiments, the bicistronic lentiviral system pLM-OSKM (Papapetrou et al. 2009) (plasmids #22240, #23242, #23243, #23244), plus the envelope psPAX and packaging pMD2G plasmids (#12259 and # 12260) were used. BC human cell lines MCF-10A (ATCC Cat# CRL-10317, RRID:CVCL_0598), MCF-7 (ATCC Cat# HTB-22, RRID:CVCL_0031) and Hs578T (ATCC Cat# CRL-7849, RRID:CVCL_0332) were a gift from Dr. Luthy’s lab, and T47D (ATCC Cat# HTB-133, RRID:CVCL_0553), BT474 (ATCC Cat# CRL-7913, RRID:CVCL_0179), MDA-MB-231 (ATCC Cat# CRL-12532, RRID:CVCL_0062), MDA-MB-468 (ATCC Cat# HTB-132, RRID:CVCL_0419) and HCC70 (ATCC Cat# CRL-2315, RRID:CVCL_1270) were a gift from Dr. Elizalde’s lab. BT474 were authenticated by the Human Cell Line Authentication STR Profiling Service from the Johns Hopkins University with the GenePrint 10 System (Promega). HepG2 (ATCC Cat# HB-8065, RRID:CVCL_0027) were a gift from Dr. Galignana’s Lab. The embryonic stem cell line WA-09 (RRID:CVCL_9773), the induced Pluripotent Stem Cells (iPSCs) (Questa et al. 2016) and the human Dermal Fibroblasts (hDFs) were provided by Dr. Leonardo Romorini and the Glioma CSCs panel (Videla Richardson et al. 2016) by Dr. Guillermo Videla Richardson. Culture media and supplements are described in Supplementary Table 1. All cells were maintained at 37 °C in a humidified 5% CO_2_ atmosphere. Transfection of MCF-7 cell line was performed with FuGENE^®^ HD (Promega; Madison, WI, USA) using a 3:1 reagent/DNA ratio. The dCas9-VP160 plasmid was transfected at a mass ratio of 1:1 to the cloned sgRNA expression plasmid in MCF-7/CR cells or to the empty sgRNA plasmid in transfection control (MCF-7/TC) cells.

### Mammosphere and extreme limiting dilution assay (ELDA)

Sphere-forming assays and ELDA were performed as previously described (Manuel Iglesias et al. 2013, Hu and Smyth 2009). Briefly, after transfection, decreasing concentrations of cells (300, 100, 30 and 10) were seeded in 200 µL of sphere media/well in 96-well culture plates, previously treated with Poly (2-hydroxyethyl methacrylate) (Sigma). Each condition was assayed in replicas and after 7–10 days of culture, the frequency of stem/progenitor cells in the cultures was calculated with the online web tool from the *Walter and Eliza Hall Institute of Medical Research Bioinformatics Division*. Sphere media was composed of DMEM/F12 (Gibco) supplemented with 2% B27, 1% glutamin, 0.1% methylcellulose, 20 ng/µL Epidermal Growth factor (EGF) and 20 ng /µL basic Fibroblast Growth Factor (bFGF).

### RNA extraction, reverse transcription and quantitative real-time PCR (qRT-PCR)

Total RNA was extracted with the TriReagent kit (MRC Inc, Cincinnati, OH, USA), according to manufacturer’s instructions. Concentration and quality of the RNA was measured with Nanodrop 2000 (Thermo Scientific, Waltham, MA, USA). The Easy Script First-Strand c-DNA Synthesis Supermix (Transgen Biotech, Beijing, China) and the Applied Biosystems Power SYBR^®^ Green PCR Master Mix (Thermofisher, Waltham, MA, USA) were used in an Applied Biosystems 7500 thermocycler. Data were normalized to glyceraldehyde-3-phosphate dehydrogenase (GAPDH) or ribosomal protein L7 (RPL7) and calculated by the 2^−DDct^ method. Data were analyzed by one-way ANOVA, followed by Dunnett’s multiple comparison test (vs MCF-10A). Primer sequences are listed in Supplementary Table 2.

### Statins activation and treatment

Simvastatin and Lovastatin were a gift from Dr. Juan Carlos Calvo and were activated according to previous reports (Liang et al. 2006, Dong et al. 2009). For treatment with statins, cells were seeded at 1–3 × 10^3^ cells/ well in 96-well plates and allowed to attach. Cells were treated with 10 to 40 µM of statins or drug vehicle and left at 37 °C in a humidified 5% CO_2_ atmosphere. After 48 h, cell viability was assessed using the CellTiter 96^®^ AQ_ueous_ One Solution Cell Proliferation assay (MTS) (Promega Corporation, WI, USA), following the manufacturer’s instructions. The absorbance was measured at 490 nm using a microplate reader (Thermo Scientific).

### Lentivirus production

For the production of 2nd-generation lentiviral vectors for reprogramming (Papapetrou et al. 2009), calcium chloride (CaCl_2_) transfection of Hek-293 T cells was used. Briefly, Hek-293 T cells were seeded onto 10 cm Petri dishes. The day of transfection, CaCl_2_, packing and envelope plasmids DNA (3:1:2, w:w:w) and water up to 500 µL were mixed, and then, 500 µL of Hepes buffered saline was added dropwise and incubated at RT for 20 min. Next, the transfection mix was added to the cells and incubated at 37 °C for 16 h. Fresh medium was added for an additional 4 h, and then, viral supernatants were collected, filtered with 0.45 µm pore size and stored at − 80 °C.

### Cell reprogramming

MCF-7 cells were seeded in 6-well plates and incubated in the presence of viral supernatants and 10 μg/mL polybrene (Hexadimethrine Bromide, Sigma) for 16–20 h. Next, viral supernatants were changed for fresh medium. Six days after infection, 100.000 were plated onto an irradiated Mouse Embryonic Fibroblasts (irrMEFs) feeder layer and the medium changed to fresh human stem cells medium [DMEM-F12 (Gibco), 20% Knock-Out Serum Replacement (Gibco), 1% de PenStrepto (Gibco), Glutamax 2 mM (Gibco), 1X Beta–mercaptoethanol (Gibco), non-essential aminoacids 0.1 mM (Gibco) and bFGF/mL 4 ng/mL (Gibco)]. After 20–40 days the colonies with the adequate morphology were manually picked and seeded onto irrMEFs for further expansion and characterization.

### Statistical analysis

Statistical significance was calculated using the software GraphPad Prism^®^ 6.00 (GraphPad Software Inc., San Diego, CA). Comparisons were performed by two-tailed unpaired t-test for two groups and one- or two-way ANOVA (as indicated in the experiments) followed by Dunnett’s as the post hoc test for more than two groups. Results were considered significant at *p* < 0.05 and are expressed as percentages relative to the control group, sample or cell line. Data are shown as the mean ± SEM of three independent experiments (*n* = 3) unless stated otherwise.

## Results

### Expression of several MVA genes is associated with worse prognosis in BC patients

**Fig. 1:**
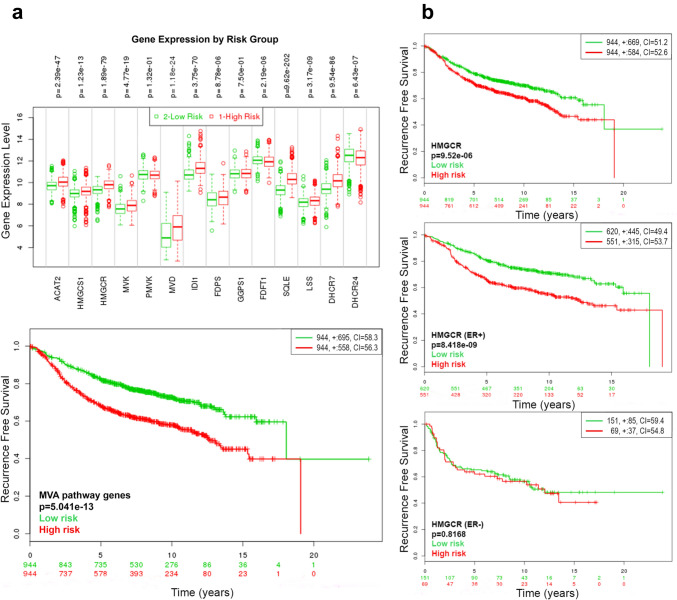
The MVA pathway is associated with worse outcome in BC patients. Box plots and KM curves of BC patients classified according to the combined expression levels of several MVA pathway genes **(a)**. KM curves according to HMGCR expression in all (*top*), ER^+^ (*center*) and ER^−^ (*bottom*) samples **(b)**. Red and Green box plots and curves denote high- and low-risk groups, respectively. KM curves for risk groups and p-value of the log-rank test are shown. Red and green numbers below horizontal axis of KM curves represent the number of individuals not presenting the event of the corresponding risk group. The number of individuals, the number of censored samples (shown as “+” marks) and the concordance index (CI) of each risk group are shown in the top-right insets. ACAT2: Acetyl-CoA acetyltransferase; HMGCS1: Hydroxymethylglutharyl Acethyl-CoA synthase 1; HMGCR: 3-hydroxy-3-methylglutaryl-coenzyme A reductase; MVK: Mevalonate kinase; PMVK: Phosphomevalonate kinase; MVD: Mevalonate pyrophosphate decarboxylase; IDI1: Isopentenyl diphosphate Delta isomerase 1; FDPS: Farnesyl diphosphate synthase; GGPS1: Geranylgeranyl pyrophosphate synthase; FDFT1: Squalene synthase; SQLE: Squalene epoxidase; LSS: Lanosterol synthase; DHCR7: 7-dehydrocholesterol Reductase and DHCR24: Delta(24)-sterol Reductase

To assess the impact of HMGCR and several members of the MVA pathway on the recurrence-free survival (RFS) of BC patients, a compiled database was analyzed with the web application SurvExpress. As shown in Fig. 1a, high expression of 11 out of the 14 genes analyzed (ACAT2, HMGCS1, HMGCR, MVK, MVD, IDI1, FDPS, GGPS1, SQLE, LSS and DHCR7), along with low expression of PMVK, FDFT1 y DHCR24, a sub-set of BC patients with high risk and a shorter RFS. In particular, HMGCR expression was associated with a decreased RFS with a hazard ratio = 1.43 (CI 95% 1.22–1.67; *p* = 9.52 e-6). Interestingly, upon stratification of HMGCR KM plots on the basis of estrogen receptor (ER) status, HMGCR was associated with a lower RFS in ER-positive patients, with a hazard ratio = 1.78 (CI 95% 1.46–2.16; *p* = 8.41 e-6), whereas there was no association in the ER-negative group (Fig. 1b).

### Differential expression of HMGCR and statin sensitivity in cancer cell lines

**Fig. 2:**
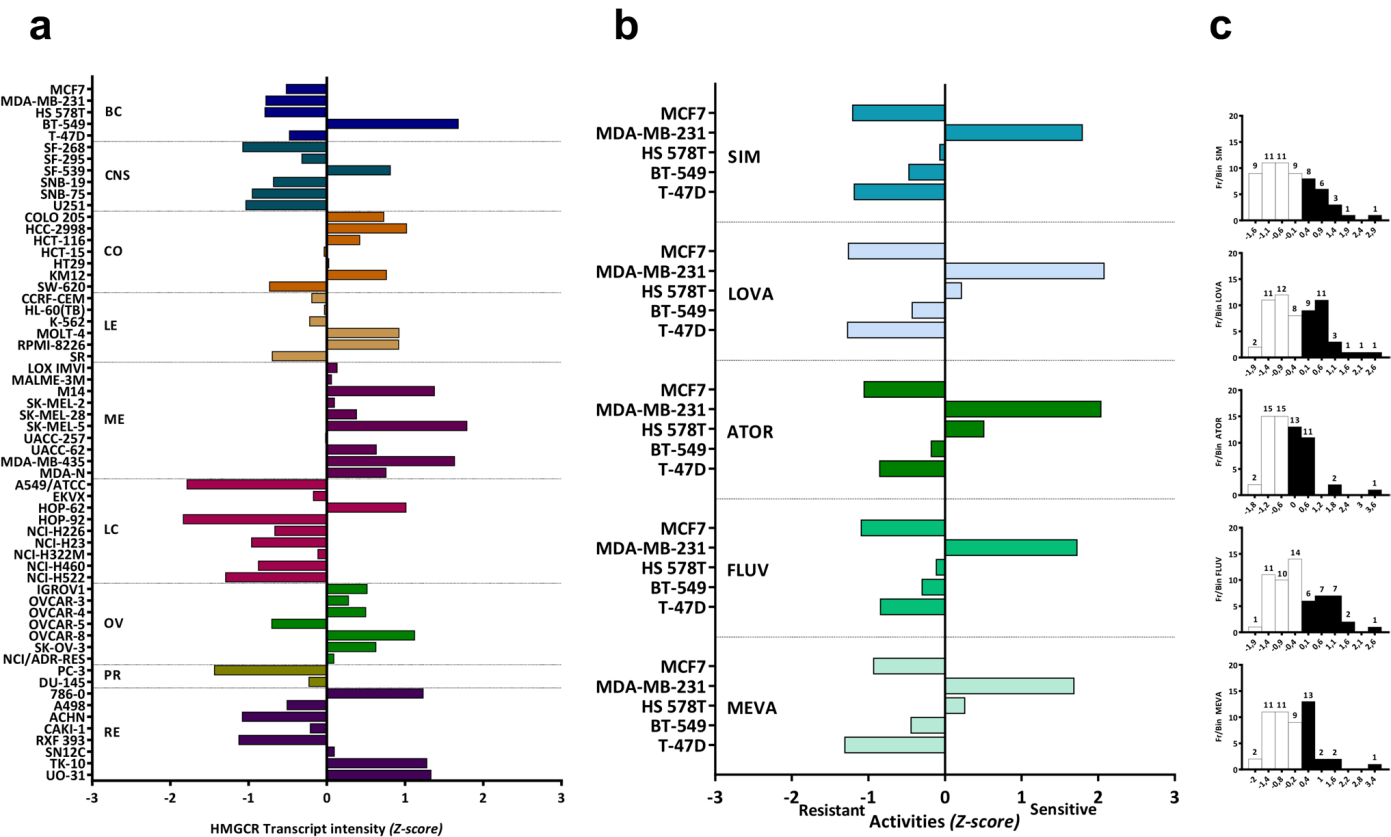
Relative transcript expression and drug activity levels in the NCI-60 panel. HMGCR expression across the NCI-60 cancer panel. BC: Breast; CNS: Central Nervous System; CO: Colon; LE: Leukemia; ME: Melanoma; LC: Lung Cancer; OV: Ovarian Cancer; PR: Prostate; RE: Renal **(a)**. Response to different statins (GI50%) in BC cell lines **(b)** and in the complete NCI-60 panel (**c)**. Data are visualized as Z-scores (**a** and **b**). In the histograms (**c**), each bin is determined by the cell line response to the drugs (x-axis). Negative values indicate resistance (white) and positive ones indicate sensitivity (black) to statins. Above the bars is indicated the number of cell lines per bin (frequency at which a particular response occurs, y-axis). SIM: Simvastatin; LOVA: Lovastatin; ATOR: Atorvastatin; FLUV: Fluvastatin; MEVA: Mevastatin

**Fig. 3:**
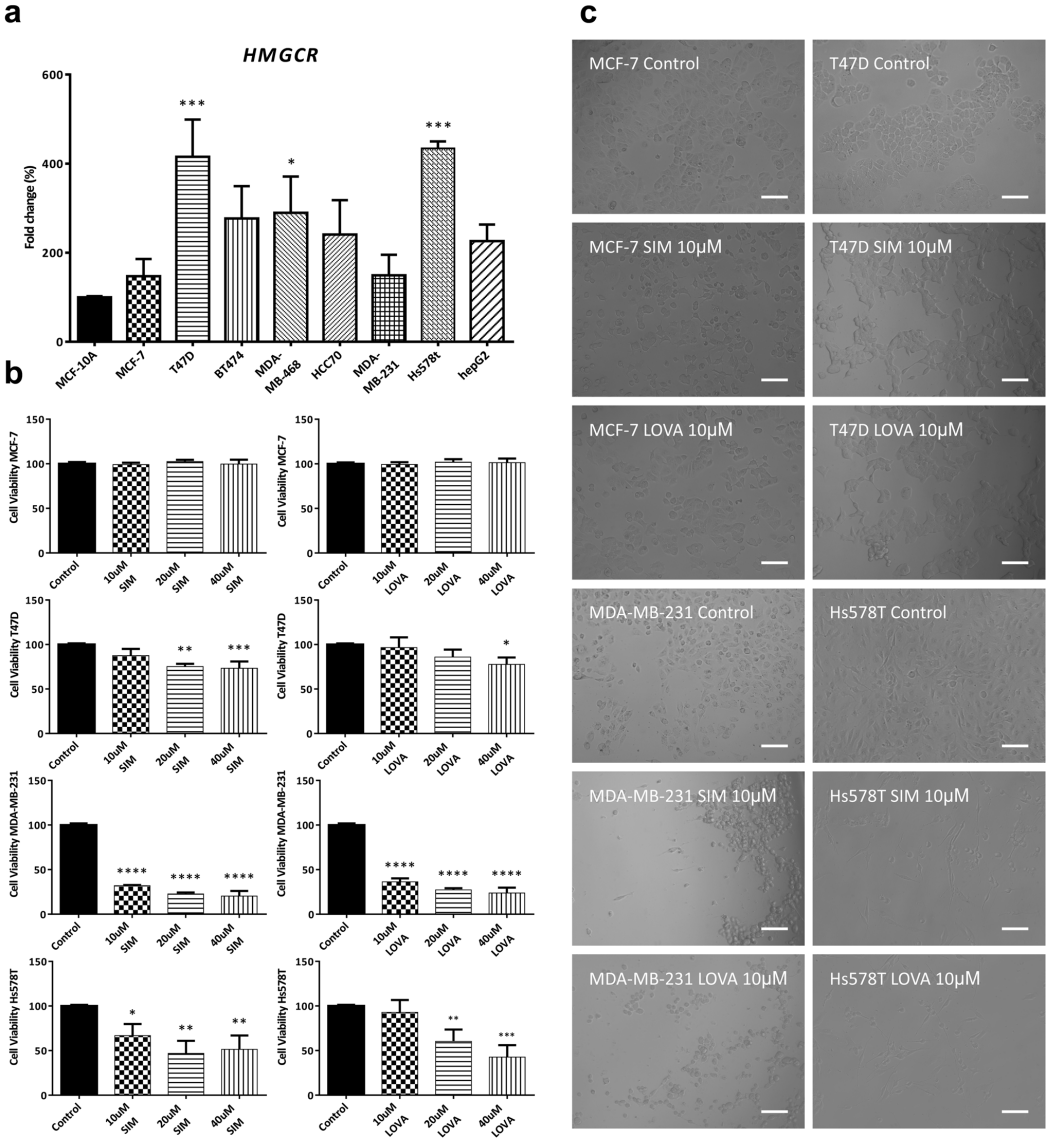
Expression of HMGCR is variable and response to statins subtype-dependent in BC. HMGCR expression was analyzed by RT-qPCR in Luminal (MC-7, T47D, BT474) and triple-negative (MDA-MB-468, HCC70, MDA-MB-231, Hs578T) BC cell lines. Hepatocellular carcinoma hepG2 cell line was used as positive control and the immortalized non-transformed mammary epithelial line MCF-10A as negative control. Data were analyzed by one-way ANOVA, followed by Dunnett’s multiple comparison test (vs MCF-10A) **(a)**. Viability of MCF-7, T47D, MDA-MB-231 and Hs578T cell lines after 48 h treatment with SIM, LOVA or vehicle (Control). Data were analyzed by one-way ANOVA, followed by Dunnett’s multiple comparison test (vs Control; *p < 0.05, **p < 0.01, ***p < 0.001, ****p < 0.0001) **(b)**. Representative images of MCF-7, T47D, MDA-MB-231 and Hs578T cell lines treated with 10 µM SIM, 10 µM LOVA or vehicle (Control). Scale bar: 100 µm **(c)**. SIM: Simvastatin; LOVA: Lovastatin

In silico analysis indicate variability in HMGCR gene expression across cancer types. In this regard, cell lines derived from melanoma and ovarian cancer showed increased levels, whereas 4 out of the 5 BC cell lines showed lower HMGCR expression when compared to the mean of the NCI-60 panel (Fig. 2a). Analysis of statin response of BC cell lines to several statins (SIM, LOVA, ATOR, FLUV and MEVA) with Cell Miner showed a general resistance to these drugs in the luminal cell lines (MCF-7, T47D) and a variable response of the triple-negative breast cancer (TNBC) cell lines (Hs578T, MDA-MB-231). Of note, the BC cell line with the highest levels of HMGCR, BT-549, consistently showed statin resistance, indicating that additional factors other than the expression of HMGCR might be regulating statins sensitivity in BC (Fig. 2b). Irrespectively of the tissue of origin, most cell lines across the NCI-60 panel were, to some degree, resistant to statins (Fig. 2c). Of interest, SIM appears to be the strongest drug, since 67.8% (40 out of 59) of the cell lines analyzed displayed resistance to treatment. The variability in HMGCR expression regardless of BC subtype and higher resistance to statins in luminal models was also corroborated in vitro in a panel of BC cell lines comprising representative luminal (MC-7, T47D, BT474) and TNBC models (MDA-MB-468, HCC70, MDA-MB-231, Hs578T) (Fig. 3a). Hepatocellular carcinoma hepG2 cell line was used as positive control and the immortalized non-transformed mammary epithelial line MCF-10A as negative control. To assess the sensitivity of BC cells to statins, viability of MCF-7, T47D, MDA-MB-231 and Hs578T cell lines was analyzed (Fig. 3b). In accordance with results from Cell Miner, both luminal BC models (MCF-7, T47D cells) showed resistance to SIM and LOVA, whereas TNBC models (MDA-MB-231 and Hs578T cells) were highly sensitive. Moreover, also in accordance with the in silico data, MDA-MB-231 cells were the most sensitive to both statins. These results further indicate that additional factors other than HMGCR (possibly BC origin and/or differentiation state) may be regulating statins response.

### Generation of a HMGCR-overexpressing system with stem-like traits in BC

**Fig. 4:**
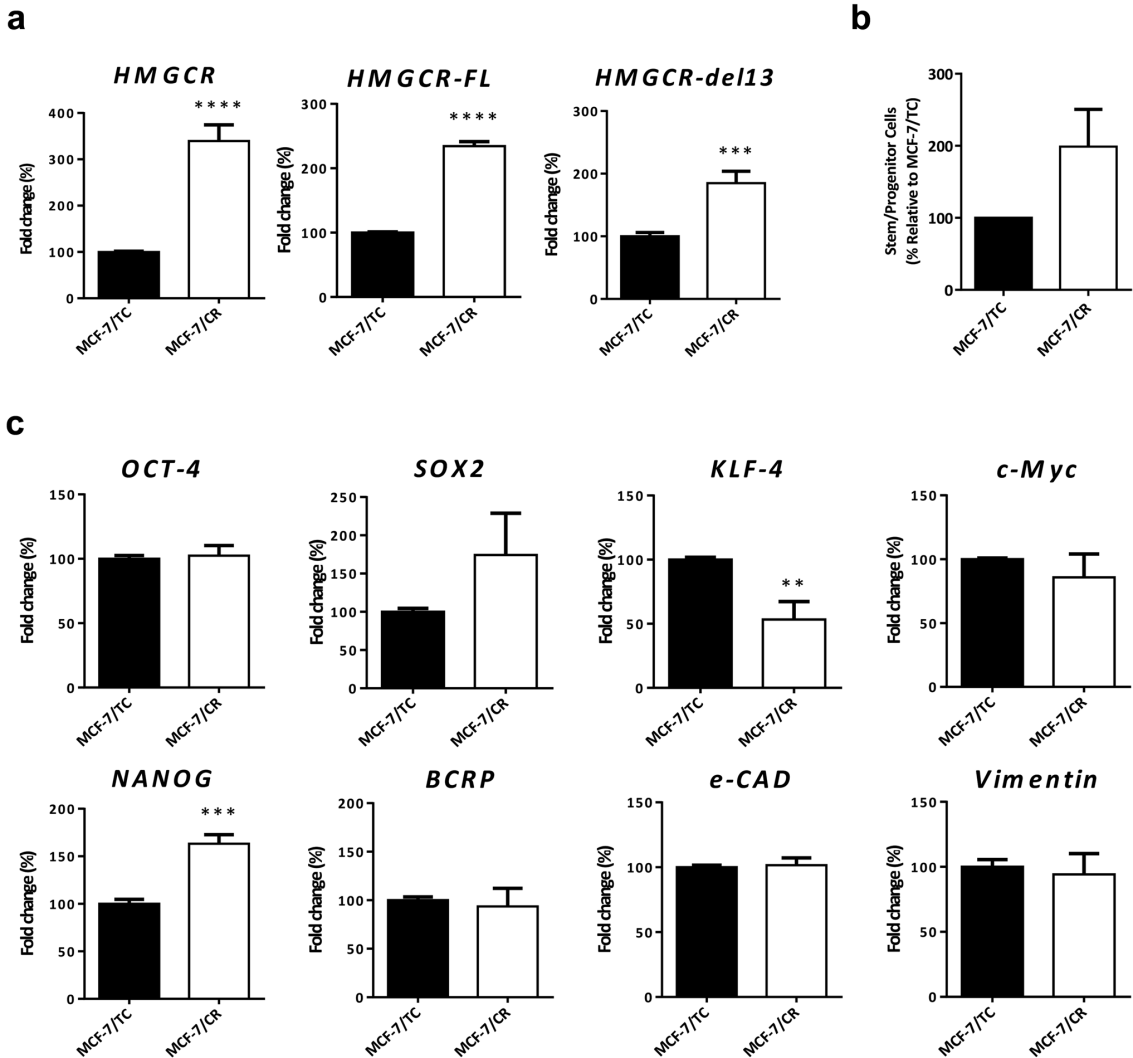
Generation on an HMGCR overexpression system with stem-like traits in MCF-7 cell line. Total HMGCR **(a)** and isoforms HMGCR-FL and HMGCR-del13 **(b)** were analyzed by RT-qPCR in MCF-7 cell line 48 h following transfection with the CRISPRon system. Quantification of mammosphere frequency in MCF-7/TC and MCF-7/CR cells **(c)**. Pluripotency and breast CSC markers (OCT4, SOX2, KLF4, c-MYC, NANOG, BCRP, ECAD and Vimentin) analyzed by RT-qPCR **(d)**. Data were analyzed by unpaired two-tailed t-test (vs MCF-7/TC; **p < 0.01, ***p < 0.001; ****p < 0.0001)

To better understand the effects of endogenous overexpression of HMGCR in BC progression, differentiation state and statins sensitivity, we developed a HMGCR overexpression model in MCF-7 BC cells. Total HMGCR mRNA levels were assessed by qRT-PCR up to 6 days post-transfection (Supplementary Fig. S2a). The time for analysis was set at 48 h and found significantly increased (more than threefold) HMGCR total levels in MCF-7/CR cells. HMGCR has two isoforms, one full length (FL-HMGCR) and one with a deletion in the exon 13 (DL13-HMGCR). We observed that the CRISPRon system significantly increased the levels of both isoforms (FL: more than twofold and DL13: more than 1.5-fold) (Fig. 4a). At the protein level, HMGCR was also increased (Supplementary Fig. S2b). To assess the appearance of stem cell-like traits in MCF-7/CR cells, we analyzed the expression of the canonical “Yamanaka Factors” OCT4, SOX2, KLF4 and c-MYC and the stem cell-related genes NANOG and ECAD and found a significant up-regulation of NANOG (1.5-fold), a moderate up-regulation of SOX2 and a significant down-regulation of KLF4 (1.5-fold). OCT4, c-MYC, BCRP, Vimentin and ECAD were not altered (Fig. 4c). The mammosphere formation assay was performed and quantified by the limiting dilution assay and statistical analysis with the specialized software as described. We found that MCF-7/CR cells had a higher frequency of mammosphere formation when compared to MCF-7/TC cells (Fig. 4b). The expression of markers more directly related to a breast CSC state was also assessed, and we found small increases in CD44^high^/CD24^negative/low^ and CD133^+^ populations in MCF-7/CR cells, although these changes were not statistically significant (Supplementary Fig. S2d and e). Finally, we found a decrease in cell adhesion and concordant increase in cell migration in MCF-7/CR cells, while no difference in proliferation was detected (Supplementary Fig. S2f).

### HMGCR is associated to fully reprogrammed stem cell states

**Fig. 5:**
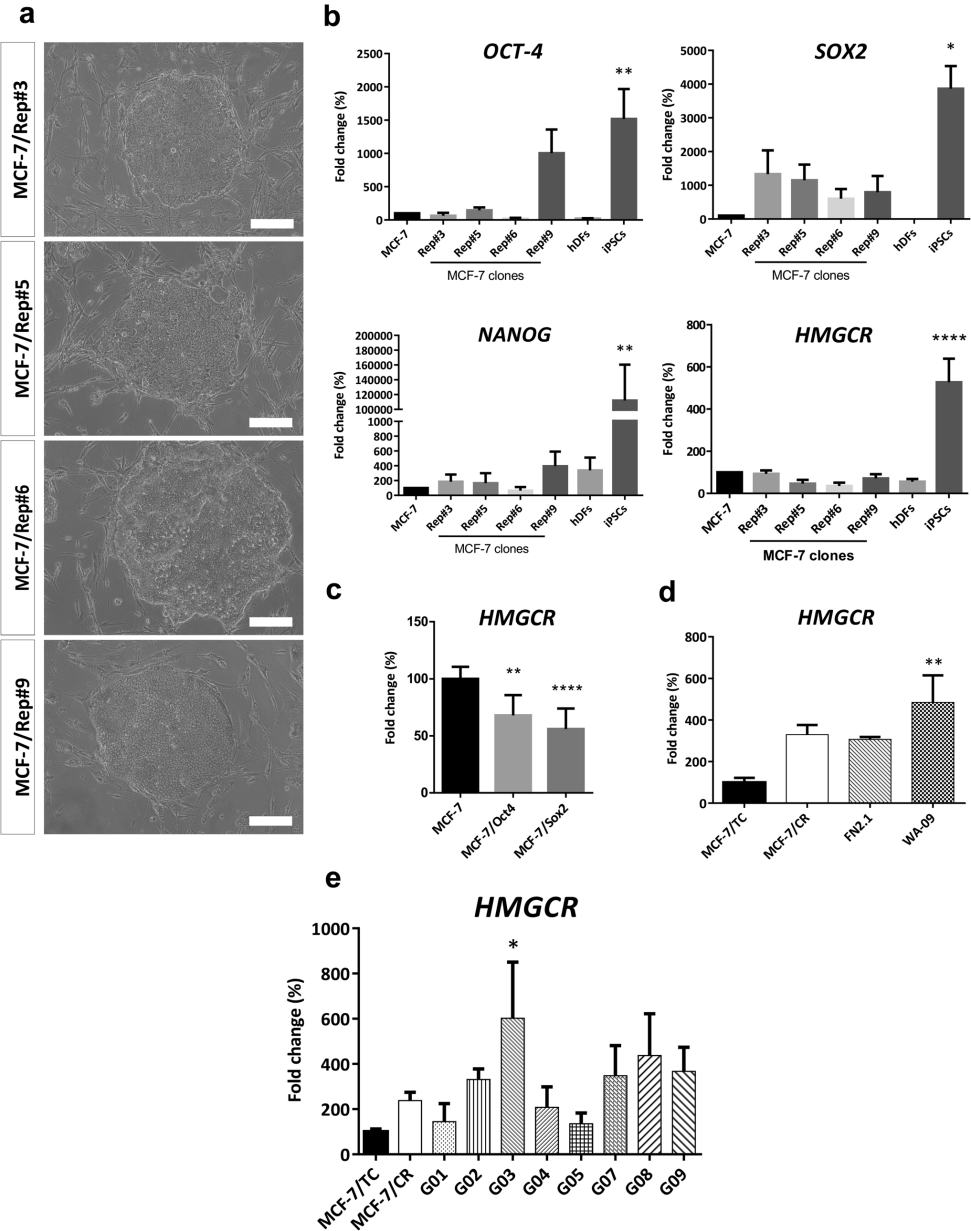
Reprogramming of MCF-7 cells. Representative images of the clones obtained upon transduction of MCF-7 cells with the lentiviral system pLM-OSKM. Scale bar: 200 µm (**a**). Initial characterization by qRT-PCR of OCT4, SOX2, NANOG and HMGCR expression of MCF-7/Rep clones, parental cell line and positive (iPSCs) and negative (hDFs) controls. Scale bar: 100 µm. hDFs, human dermal fibroblasts (**b**). HMGCR expression in MCF-7 cells separately transduced with the pluripotency factors OCT4 and SOX2 (**c**), in pluripotent stem cell lines **(d)** and in a panel of glioblastoma-derived CSCs **(e)** compared to the HMGCR overexpression system generated with MCF-7 cells. Data were analyzed by one-way ANOVA, followed by Dunnett’s multiple comparison test (vs MCF-7 in b and c and vs MCF-/TC in d and e; *p < 0.05, **p < 0.01, ****p < 0.0001)

Taking into account that MCF-7/CR cells showed increased expression of the stemness markers NANOG and SOX2, it was tempting to speculate that HMGCR might be associated to the acquisition of stemness in BC. With this in mind, we set out to assess the expression of HMGCR during the reprogramming of the luminal BC cell line MCF-7 with defined factors (OCT4, SOX2, KLF4 and c-MYC). Upon infection and seeding onto an irrMEFs feeder layer, we obtained colonies with different morphologies, picked those with defined edges, comprising compact, small cells and stablished four clones, termed MCF-7/Rep #3, #5, #6 and #9 (Fig. 5a). Initial characterization performed by qRT-PCR showed increased expression of SOX2 and low expression of NANOG in all clones and one clone, MCF-7/Rep #9, showed increased expression of OCT4 when compared to parental MCF-7 cells. Human Dermal Fibroblasts (hDFs) and iPSCs were included as negative and positive controls, respectively (Fig. 5b). Immunofluorescent detection of pluripotency markers was performed at the same passages in MCF-7/Rep clones #3 and #9, further confirming the results obtained at the transcriptional level for SOX2, NANOG and OCT4. Neither the parental cell line MCF-7 nor the clones showed expression of the cell surface markers TRA-1-60 and SSEA4 (Supplementary Fig. S3a). Furthermore, alkaline phosphatase activity was not observed in any of the MCF-7/Rep clones (Supplementary Fig. S3b). Finally, we analyzed HMGCR expression in all the MCF-7/Rep clones and observed levels comparable to those expressed in parental MCF-7 cells (Fig. 5b). To further confirm that the core pluripotency factors SOX2 and OCT4 do not upregulate HMGCR expression, we transduced MCF-7 cells with pLM-OCT4 or pLM-SOX2 and found decreased HMGCR levels (Fig. 5c). Next, we addressed whether HMGCR was associated with established stem cell phenotypes, in both transformed and non-transformed cell lines. We also included in this analysis our stem-like model MCF-7/CR. With this purpose, we assessed HMGCR expression at the transcriptional level in WA-09, iPSCs and eight cell lines enriched in CSCs from glioblastomas (termed G01, G02, G03, G04, G05, G07, G08 and G09). Both pluripotent cell lines showed increased levels of HMGCR when compared to MCF-7/TC cells, and interestingly, the iPSCs showed levels comparable to those of the MCF-7/CR cells (Fig. 5d). The glioblastoma CSC panel showed high variability in HMGCR levels but increased when compared to MCF-7/TC cells (Fig. 5e). Collectively, these results suggest that HMGCR expression may be an important trait of fully reprogrammed phenotypes in non-malignant cells, and a tumor type-dependent trait in highly undifferentiated malignant cells.

## Discussion

Accumulating evidence indicates that stem-like states could be generated via three critical mechanisms, including gene transfer, genomic instability, and microenvironment alteration (Azizidoust et al. 2023). The latter is particularly important since the biomechanical nature of the extracellular matrix (ECM) is able to regulate cell fate in physiological (embryonic development) and pathological (cancer and fibrotic diseases) situations (Li et al. 2020). For instance, ECM stiffness in fibrotic tissues activates Yes-associated protein (YAP) and the transcriptional coactivator with PDZ-binding motif (TAZ), key effectors of the Hippo pathway, in epithelial cells, thus promoting cell proliferation, survival and stemness (Noguchi et al. 2018). Interestingly, YAP/TAZ are targeted by statins in different tumor types (Sethunath et al. 2019; Uemura et al. 2023; Benhammou et al. 2023). Therefore, understanding the intrinsic, microenvironmental and metabolic cues that enable the reprogramming of cancer cells into stem-like states could help unravel cancer pathogenesis and therapy resistance, thus contributing towards the development of more effective treatments. In the present work, we aimed to elucidate the relationship between cholesterol metabolism, statin sensitivity and the induction of stemness in BC.

Previous studies, in which HMGCR protein levels were associated with favorable clinicopathological characteristics (Borgquist et al. 2008; Gustbée et al. 2015), have been challenged by gene expression data, although a more recent study again associates HMGCR expression with better clinical parameters (Yulian et al. 2023), proving that the relationship between HMGCR and prognosis in BC is still controversial. To this regard, our analyses in silico indicated that overexpression of several members of the MVA pathway, including HMGCR, is associated with lower RFS in BC. Supporting our findings, HMGCR expression was shown to be associated with poor prognosis and decreased survival in BC patients, in PDX from TNBC and with more aggressive tumor characteristics such as higher histological grade, high Ki67 and ER negativity (Clendening et al. 2010; Ehmsen et al. 2019; Bjarnadottir et al. 2020). Our analysis of 60 cell lines from 12 different tumors with Cell Miner showed different expression profiles of HMGCR, which not always correlated with sensitivity to statins, suggesting that other factors may be contributing to overall statin response. Indeed, by specific analysis of the BC cell lines within the NCI-60 panel, we found a similar response of each cell line to several statins, with high sensitivity of most ER-negative cell lines and resistance in ER-positive cells. Interestingly, neither BC subtype nor statin response appears to be completely related to HMGCR expression, as we further validated in vitro. Rather, statin response seems to be associated with the proportion of mesenchymal-like cells (Dolfi et al. 2013; Brooks et al. 2015). In line with this, we found that Hs578T and MDA-MB-231 cell lines, which show a gene expression profile similar to claudin-low tumors (Prat et al. 2010) were highly sensitive to statins, whereas luminal MCF-7 and T47D cell lines, characterized by the lowest proportion of CSCs and epithelial-like bulk cells (Brooks et al. 2015) were resistant. However, statin concentrations required to eliminate statin-sensitive cells are higher than those observed in human plasma during hypercholesterolemia therapy (Ishikawa et al. 2018), highlighting the clinical need to enhance the effect of statins on cancer cells, mainly by its use as part of combination therapies. Indeed, SIM has been shown to improve the response to neoadjuvant therapy with fluorouracil, adriamycin, and cyclophosphamide (FAC) in patients with locally advanced BC, although the exact molecular mechanisms underlying the benefits of combining SIM and chemotherapy are not fully known (Yulian et al. 2021). Moreover, the same authors recently reported greater potential of statins in patients with locally advanced BC with low or no HMGCR expression (Yulian et al. 2023).

High concentrations of ATOR or SIM decreased the expression of pluripotency markers in human iPSCs and ATOR impaired the formation of teratomas in immunodeficient mice (Nakashima et al. 2018), suggesting that statins may function as “tissue sweepers” of potentially rogue stem cells, ultimately being able to target undifferentiated and tumorigenic cells. Therefore, we used a CRISPR-CAS9 transcriptional activation system (CRISPRon) to determine whether HMGCR could act as a facilitator for the acquisition of stem-like states, considering: 1- MCF-7 cells expressed low levels of HMGCR; and 2- being a luminal BC cell model, MCF-7 contain a population of mostly epithelial tumor bulk cells with a low frequency of CSCs. In our system, induction of HMGCR increased the sensitivity of MCF-7 cells to high concentrations of SIM, whereas there were no effects on the response to LOVA. We decided then to explore functional indicators of malignancy such as the loss of cell adhesion and migration upon HMGCR overexpression and found that MCF-7/CR cells showed significantly higher migration ability and a concordant decrease in cell adhesion. These results are in line with the in vitro evidence of a distinct role of HMGCR in the transformation and migration of several tumor types, including BC (Clendening et al. 2010; Singh et al. 2015; Ehmsen et al. 2019). Resistance to anoikis is another functional trait that defines CSC-like populations, thus, we evaluated mammosphere formation ability in MCF-7/CR cells. Supporting our data, others have found that MVA pathway genes were overexpressed in mammospheres from TNBC (Ginestier et al. 2012). Since HMGCR seems to promote the growth of cells in undifferentiated states, we analyzed the expression of main pluripotency regulators and found that NANOG was significantly increased in MCF-7/CR cells. Strong expression of NANOG has been shown to be an indicator of poor prognosis in BC (Nagata et al. 2014), and further confirmed in TNBC (Nagata et al. 2017). A more recent work also supports the idea of a subtype-dependent role of pluripotency markers in BC, since OCT4, SOX2 and NANOG were overexpressed in Her2 + BC tumors and associated with shorter overall survival (Yang et al. 2018). In vitro, tamoxifen-resistant T47D cells showed increased mammosphere formation ability, altered mammosphere morphology, and overexpression of OCT4 and NANOG, further enforcing the role of these key pluripotency markers in BC progression and drug resistance (Rodriguez et al. 2023). We also observed a significant decrease of KLF4 in MCF-7/CR cells, interestingly, high expression of this marker was associated with better disease-free survival in BC (Nagata et al. 2014) and overall survival in TNBC (Nagata et al. 2017). In addition, we found a moderate increase in SOX2 expression in MCF-7/CR cells, suggesting other pluripotency networks may also interplay with HMGCR in stem-like states. Conversely, HMGCR was expressed at high levels in well-established pluripotent stem cell models as well as CSC lines from glioblastoma, suggesting that HMGCR might be a marker of acquired stem phenotypes in transformed and non-transformed cells. Furthermore, following reprogramming of MCF-7 cells, 4 reprogrammed clones were obtained (MCF-7/Rep), characterized by increased expression of SOX2, in accordance with previously published reprogrammed MCF-7 cells (Corominas-Faja et al. 2013), in which endogenous transcriptional activation of SOX2, mTOR and increased activity of lipogenic pathways were described. These findings provide additional evidence that metabolic reprogramming, and particularly, a lipogenic phenotype, is a requirement for the acquisition of stem-like states in BC (Corominas-Faja et al. 2013). However, analysis of HMGCR in MCF-7/Rep cells revealed expression levels comparable to their parental counterparts. Of note, in our experiments, NANOG was the pluripotency marker more significantly upregulated in MCF-7/CR cells but showed no significant up-regulation in MCF-7/Rep cells. Thus, the absence of HMGCR (and NANOG) expression in the reprogrammed clones may reflect the incomplete or partial reprogramming usually observed in cell lines covering a wide range of human cancers, including BC (Lin et al. 2008; Miyoshi et al. 2010; Mathieu et al. 2011; Corominas-Faja et al. 2013; Stricker et al. 2013). It may also reflect that a single tumor contains breast CSCs with distinct molecular profiles, as reviewed in Song and Farzaneh (2021), and does not exclude the role of HMGCR as a promoter of stem cell traits through certain pluripotency transcription networks, such as NANOG. Indeed, fundamental biological barriers, including cancer-specific genetic mutations, epigenetic modifications, accumulation of DNA damage and reprogramming-induced cellular senescence prevent the rewiring of most malignant cancer cells into pluripotency (Hochedlinger et al. 2004; Ramos-Mejia et al. 2012; Kim 2015). Although there are many unanswered questions, the high expression of HMGCR in pluripotent cells and CSCs models reported in this work, together with an HMGCR-induced stem-like phenotype in MCF-7 cells, suggests that this enzyme may act as a facilitator for the acquisition of stem-like states and be relevant to the maintenance of well-stablished stem cell states, possibly through the NANOG transcription network.

## Conclusion

**Fig. 6:**
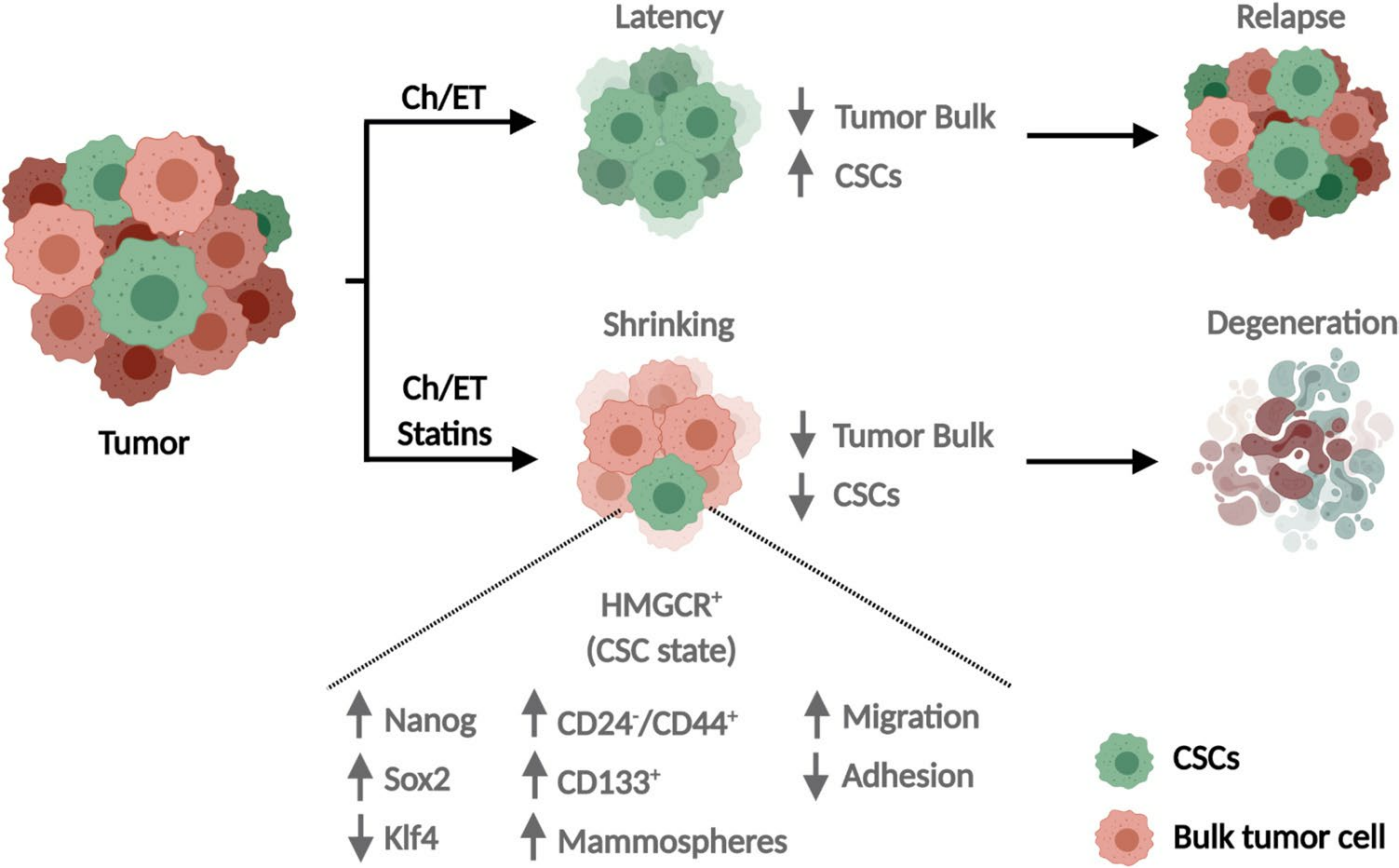
HMGCR overexpression model and proposed use of statins as adjuvant therapy to target CSCs in BC. HMGCR overexpression induces CSC-states in BC. Conventional therapy targets the bulk of the tumor while drug-resistant CSCs remain in latency and later regenerate the tumor. Addition of statins to treatment protocols may target specifically the stem compartment, contributing to tumor shrinking and degeneration, reducing metastasis, relapses and improving treatment response. Ch: Chemotherapy; ET: Endocrine Therapy. Created with BioRender.com

Here, we provide evidence that the endogenous transcriptional activation of HMGCR in cells with strong epithelial nature, such as MCF-7 cells, promotes the appearance of stem-like traits in BC. This could impact tumor initiation and progression, as well as statin sensitivity in the context of ER-positivity. Emerging as a potential alternative for the treatment of hormone-dependent and independent BC, statins may contribute to tumor shrinking, inhibition and/or delay of metastasis and relapse (Fig. 6). Overall, these results encourage additional studies to unravel the role of a still unexplored HMGCR/NANOG partnership in the metabolic reprogramming of tumors and the repurposing of statins as potential adjuvant therapies, which may prevent the generation and maintenance of stem-like, therapy-refractory states in BC.

## Supplementary Information

Below is the link to the electronic supplementary material.

Supplementary file1 (DOCX 4235 KB)

## Data Availability

The data analysed in this study are available in the following repositories: SurvExpress (http://bioinformatica.mty.itesm.mx:8080/Biomatec/SurvivaX.jsp) and Cell Miner (https://discover.nci.nih.gov/cellminer/). Other data analysed in this study are available from the corresponding author upon reasonable request.

## Abbreviations

BC: Breast cancer
CSC: Cancer stem cell
MVA: Mevalonic acid pathway
RFS: Recurrence-free survival
ER: Estrogen receptor
TNBC: Triple-negative breast cancer
iPSCs: Induced pluripotent stem cells
PBS: Phosphate buffer solution
RT: Room temperature
ON: Overnight
