# Supplementary material for: Role of hydroxymethylglutharyl-coenzyme A reductase in the induction of stem-like states in breast cancer
Source: J Cancer Res Clin Oncol. 2024 Feb 28;150(2):106. doi: 10.1007/s00432-024-05607-7 (PMC10902018; doi:10.1007/s00432-024-05607-7)
Supplement: Supplementary file 1 — Supplementary file1 (DOCX 4235 KB) [file 432_2024_5607_MOESM1_ESM.docx]

**Expression of hydroxymethylglutharyl-coenzyme A reductase induces stem-like states in breast cancer cells.**

María Paula Marks, Carla Alejandra Giménez, Luciana Isaja, Mariana Belén Vera, Francisco Raúl Borzone, Federico Pereyra-Bonnet, Leonardo Romorini, Guillermo Agustín Videla-Richardson, Norma Alejandra Chasseing, Juan Carlos Calvo, Luciano Vellón

Supporting information

**Supplementary Fig. S1: Generation of the CRISPRon system.** Sequence of the 5 probes selected from the Genome Engineering Toolbox and schematic representation of their target on the proximal region of the promoter of the human HMGCR gene **(a)**. Chemically competent *Escherichia coli* were transformed with the constructs and ampicillin-resistant clones were selected for expansion and storage. Plasmid DNA was extracted with a Midiprep kit (Quiagen) following the manufacturer’s instructions. Verification of the cloning procedure was performed by sequencing and *in silico* analysis with the softwares FinchTV (one representative image is shown) **(b)** and MEGA (sequences of digested pSPgRNA plasmid, probes, and two clones per probe are shown) **(c).**


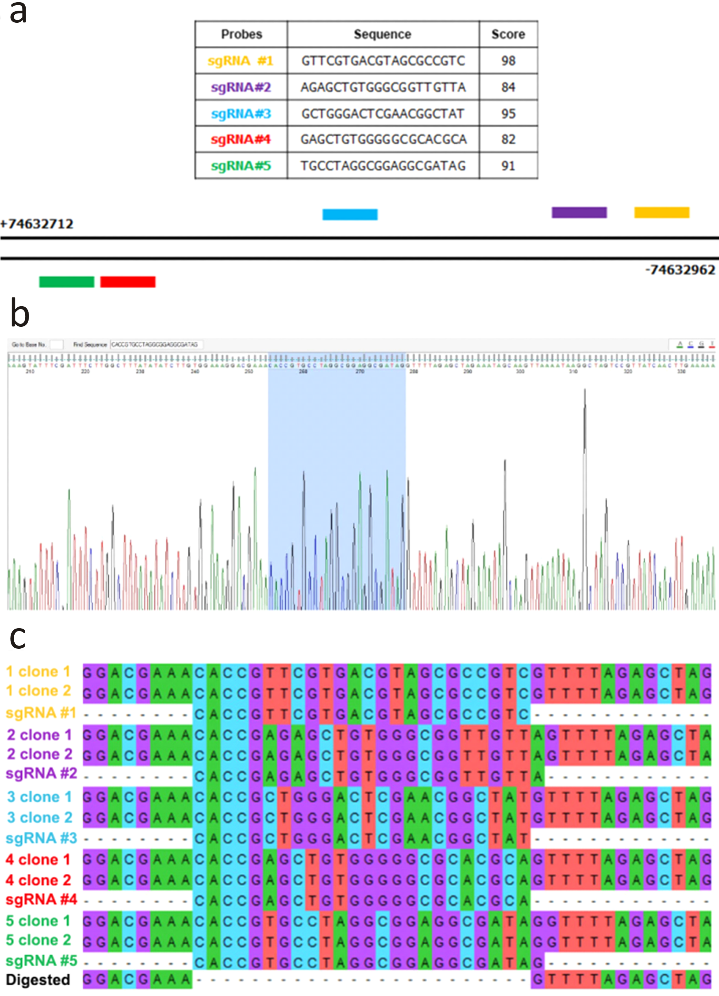


**Supplementary Fig. S2: Characterization of the CRISPRon system.** HMGCR expression was assessed by qRT-PCR at 48 h, 96 h and 6 days **(a)** and by western blot at 48 h post transfection. Here, the bands corresponding to HMGCR and the loading control (ß-tubulin) were cropped for better visualization and figure design. The samples for quantification derive from the same blots and were processed in parallel. **(b)**. Viability was assessed in MCF-7/TC and MCF-7/CR cells after 48 h treatment with 10-40 µM SIM, LOVA or vehicle (Control). Data were analyzed by two-way ANOVA, followed by Dunnett´s multiple comparison test (vs MCF-7/TC Control; *p< 0.05) **(c)**. Dot-blots and quantifications of CD44^+^/CD24^-/low^ **(d)** and CD133^+^ **(e)** populations in MCF-7/TC and MCF-7/CR cells. Adhesion, migration (n=2) and proliferation assays in MCF-7/TC and MCF-7/CR cells 48 h post transfection **(f)**. Data were analyzed by unpaired two-tailed t-test (vs MCF-7/TC; ****p<0.0001). SIM: Simvastatin; LOVA: Lovastatin.


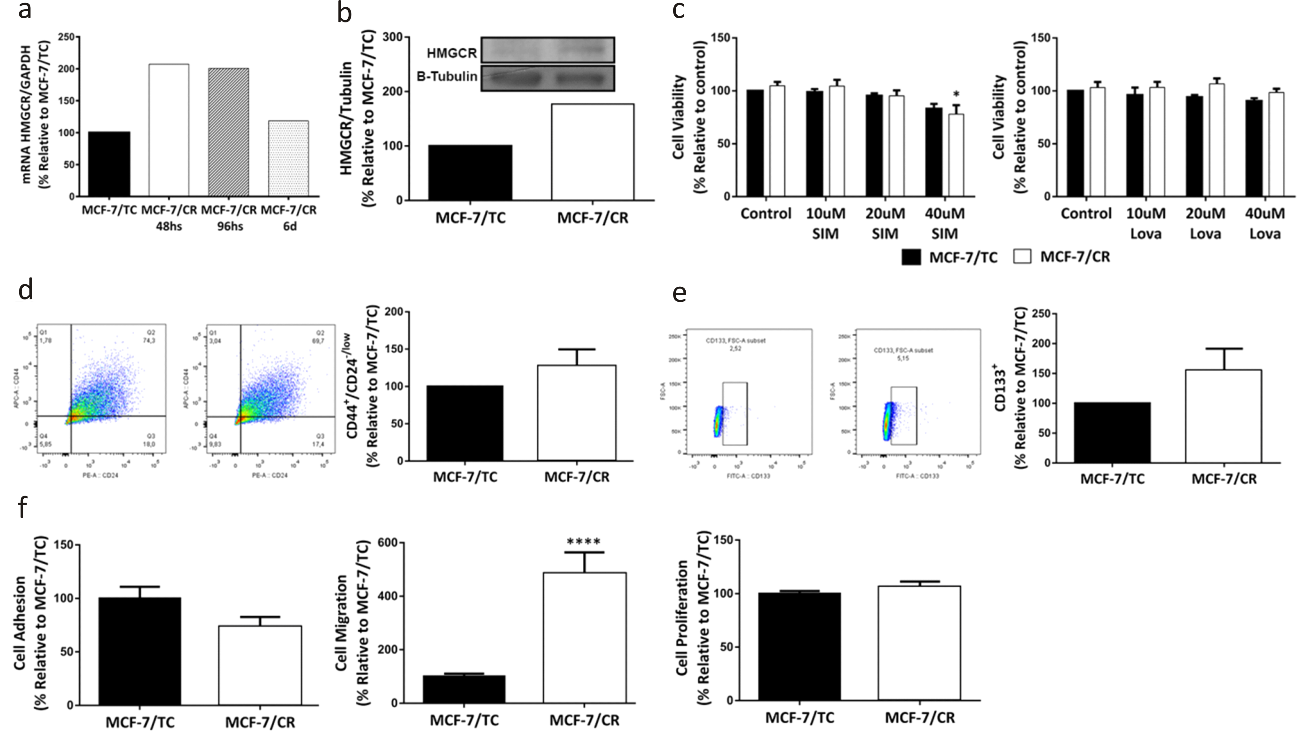


**Supplementary Fig. S3: Pluripotency markers in MCF-7/Rep cells. (a)** Immunofluorescent detection of the pluripotency markers OCT4, NANOG, SOX2, TRA-1-60 and SSEA4 in MCF-7/Rep clones #3 and #9, the parental cell line MCF-7 and an iPSCs cell line used as positive control. **(b)** Representative images of alkalyne phosphatase (AP) assays performed on MCF-7/Rep #3, #5, #6 and #9, on the iPSCs cell lines FN2.1 and FAD and in the glioma CSCs cell lines G01, G03 and G09. Positive mark is visualized as a purple staining. Scale bar: 100 µm.


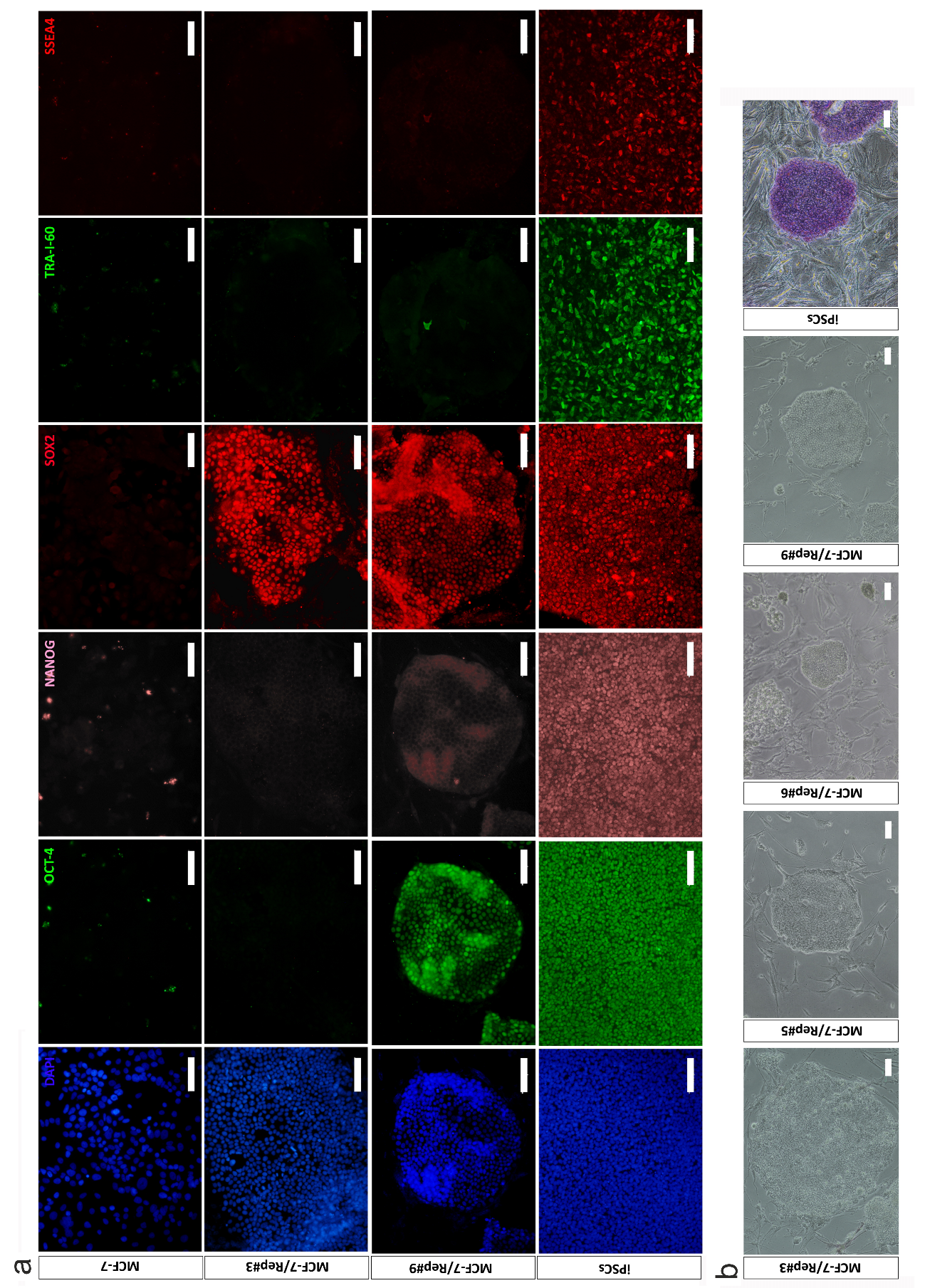


**Supporting Information**

For western blot analysis, cells were transfected as indicated and then lysed in ice using scrapers and buffer (60 mM Tris-HCl pH 6.8 and 1% W/V SDS in H_2_0d). The lysates were collected in eppendorf tubes, boiled for 3 min, vortex for 20 seg and boiled again for 7 min. Finally, tubes were centrifuged for 10 min at 300 xg and supernatants were collected in new tubes. Total protein was quantified using a Nanodrop 2000 (Thermo Scientific, Waltham, MA, USA). Proteins (60-80 µg) were loaded and separated in a 12% SDS-PAGE gel and electro-transferred to a nitrocellulose membrane (Amersham). The membrane was later blocked for 1 h with bovine serum albumin (Sigma-Aldrich, 0055 K) and then incubated with the primary antibodies ON at 4°C. The membranes were then washed, incubated with the corresponding secondary antibodies conjugated to HRP for 1:30 h and washed again. Antibody complexes were visualized by means of chemiluminescence (ECL; GE Healthcare) using the detection equipment G:BOX (Syngene, UK)**.** Band density was measured using ImageJ software (NIH, Bethesda, MD, USA). Tubulin was used as endogenous control. The antibodies used were HMGCR (1/300, Santa Cruz Biotechnology), tubulin (1/5,000; Abcam), anti-rabbit-HRP (1/6,000; Sigma) and anti-mouse-HRP (1/4,000; Sigma).

For cytometric analysis, cells were transfected as indicated and then 1.5x10^5^ cells were washed in PBS and blocked in 10% normal goat serum for 30 min at RT. Then, primary antibodies were added and incubated for 30 min in ice in the dark. The antibodies were diluted in blocking solution: 1/80 for CD44 (Biolegend) and 1/100 for CD24 (Biolegend). Cells were then washed, centrifuged at 400 xg for 5 min at 4°C, fixed in 4% PFA for 30 min and washed again. CD133 antibody (VioBright ™ 515) was diluted 1/40 in dilution buffer (2 mM EDTA and 0.5% bovine serum albumin in PBS) and added to the cells for 10 min in the dark at 4°C. Cells were then washed in buffer, centrifuged at 300 xg for 10 min, fixed in 2% PFA for 10 min at 4°C and washed again. Finally, in both assays, cells were resuspended in 100 µL and analyzed by flow cytometry. Data were acquired on a BD FACScanto™ II and analyzed using FacsDiva y FlowJo software.

Cell adhesion assay was performed as previously described with minor modifications^3^. Briefly, cells were transfected as indicated and then 6x10^4^ cells were seeded in 200 µL of complete media/well on a 96-well culture plate, followed by 90 min incubation. Afterwards, culture media was removed, and cells were washed twice with PBS, fixed with methanol for 10 min and stained with 0.5% crystal violet for 10 min. The cells were washed twice with distillated water and the dye was dissolved in a solution of 10% methanol and 5% acetic acid. Each condition was assayed in triplicate and wells without cells were used as blank. The absorbance was measured at 620 nm using a microplate reader (Thermo Scientific).

For the Transwell Migration Assay, the cells were transfected as indicated and then 1.5x10^4^ cells were seeded in 200 µL of DMEM/F12 without serum in the upper chamber of a 24-well 8.0 µm pore size Cell Culture Insert (Biofil). The bottom chamber was filled with 500 ml of DMEM/F12 10% FSB. After 15 h, inserts were washed and cells that had migrated to the bottom side of the inserts were stained with DAPI and counted in an inverted fluorescence microscope (Olympus CKX-41).

Immunofluorescent detection of pluripotency markers was performed as follows: cells were washed with PBS and fixed with 4% paraformaldehyde during 45 min. Following three washes with 0.1% bovine serum albumin (BSA, Sigma-Aldrich, 0055 K), cells were permeabilized and blocked in 0.1% BSA, 0.1% Triton-X-100 (Sigma) and 10% fetal bovine serum (Gibco) for 30 min at RT. Next, cells were incubated with the corresponding primary antibodies at 4ºC ON. Primary antibodies were then washed, and the cells incubated with the corresponding secondary antibodies for 45 min at RT. Nuclei were stained with DAPI during 15 min and washed. Images were acquired using a Nikon Eclipse inverted microscope equipped with a Nikon DXN1200F digital camera and analyzed with the EclipseNet software (versión 1.20.0 build 61). Primary and secondary antibodies: Nanog (cat # D73G4, Cell Signalling), Oct4 (cat # sc5279, Santa Cruz), TRA-1-60 (cat # sc-21705, Santa Cruz), SSEA4 (cat # sc-21704, Santa Cruz), Sox2 (cat # PA1-16968, Thermo), anti-rabbit 555 (cat # A31572, Life Technologies), anti-mouse 488 (cat # A11029, Life Technologies), anti-mouse 488 (cat # A21042, Molecular Probes), anti-mouse 555 (cat # A21424, Molecular Probes) and anti-rabbit 555 (cat # ab150074, Abcam). All antibodies were diluted in 0.1% BSA and 10% Normal Goat Serum.

Alkaline Phosphatase assays were performed using a commercial kit and according manufacturer’s instructions (cat # 86R-1KT, Sigma). Briefly, the cells were washed with PBS and then fixed with a citrate, acetone and formaldehyde solution for 30 seconds. Following one washing, the cells were stained with a diazonium salt and naphthol AS-BI alkaline solution during 15 minutes in darkness. The cells were then washed twice with dH2O and the plates dried at RT. Images were acquired using a Nikon T1-SNCP inverted microscope and visualized with a digital camera and screen (Nikon, DS-Fi2 y Nikon digital sight DS-L3, respectively).

**References:**

Dong W, Vuletic S, Albers JJ. Differential effects of simvastatin and pravastatin on expression of Alzheimer's disease-related genes in human astrocytes and neuronal cells. *J Lipid Res*. 2009;50(10):2095-2102. doi:10.1194/jlr.M900236-JLR200

Liang SL, Liu H, Zhou A. Lovastatin-induced apoptosis in macrophages through the Rac1/Cdc42/JNK pathway. *J Immunol*. 2006;177(1):651-656. doi:10.4049/jimmunol.177.1.651

Farré PL, Scalise GD, Duca RB, et al. CTBP1 and metabolic syndrome induce an mRNA and miRNA expression profile critical for breast cancer progression and metastasis. *Oncotarget*. 2018;9(17):13848-13858. doi:10.18632/oncotarget.24486

**Supplementary Table 1: Culture media composition for each cell line.** FBS: Fetal Bovine Serum; EGF: Epidermal Growth Factor; bFGF: basic Fibroblast Growth Factor; NEAA: Non-Essential Aminoacids; MEFs: Murine Embryonic Fibroblasts.

| Cell Line | Complete Medium Composition |
| --- | --- |
| MCF-7, T47D,  MDA-MB-468, Hs578T | DMEM-F12, 10% FBS, 1% L-glutamine, 2 μg/mL insulin. |
| BT474 | RPMI 1640, 10% FBS, 1% L-glutamine. |
| MDA-MB-231 | DMEM-F12, 10% FBS, 1% L-glutamine. |
| HCC70 | RPMI 1640, 10% FBS, 0.45 g/mL glucose, 1M HEPES, 100mM sodium pyruvate. |
| MCF-10A | DMEM-F12, 10% FBS, 2 μgmL insuline, 0.5 μgmL cortisol, 20 ng/mL EGF. |
| HepG2, hDFs, Hek293T, irrMEFs | DMEM-high glucose, 10% FBS, 1% L-glutamine. |
| WA-09, iPSCs, MCF-7/Rep clones | Knock Out-DMEM, 20% Knock Out-Serum Replacement, 1% L-glutamine, 2- β –mercaptoethanol, 2% NEAA and 20 ng/mL bFGF. Maintained as co-cultures in the presence of a feeder layer of inactivated MEFs. |
| Glioma CSC panel | Neurobasal media, 2 mM L-glutamine, B-27™, N-2™, 20 ng/mL bFGF, 20 ng/mL EGF, 2mM NEAA, 50 U/mL penicillin/streptomycin. |

**Supplementary Table 2: Primer sequences used for qRT-PCR.**

| Primers | Sense | Antisense | Annealing |
| --- | --- | --- | --- |
| HMGCR | GTTCTGAACTGGAACATGGGC | TTCATCCTCCACAAGACAATGC | 60°C |
| HMGCR-FL | TGCAGAGCAATAGGTCTTGGTG | TCGAGCCAGGCTTTCACTTC | 60°C |
| HMGCR-del13 | GGGATTATAATTACTCCTTGCTTGGTG | TCGAGCCAGGCTTTCACTTC | 60°C |
| OCT4 | GGTCCGAGTGTGGTTCTGTA | GGAAAGGGACCGAGGAGTAC | 60°C |
| NANOG | ATGCAACCTGAAGACGTGTG | AGGCTCCAACCATACTCCAC | 60°C |
| SOX2 | AGCATGGAGAAAACCCGGTACGC | CGTGAGTGTGGATGGGATTGGTGT | 60°C |
| c-MYC | TCAAGAGGTGCCACGTCTCC | TCTTGGCAGCAGGATAGTCCTT | 60°C |
| KLF4 | TGGGGTTTTGGGTTTTGGCTTCG | CGCCAGGTGGCTGCCTCATT | 58°C |
| BCRP | CAGGTCTGTTGGTCAATCTCACA | TCCATATCGTGGAATGCTGAAG | 60°C |
| Vimentin | CACTCCCTCTGGTTGATAC | GTGATGCTGAGAAGTTTCG | 58°C |
| ECAD | TTTGCTAAGTAAGTCCAG | TTTGCTAATTCTGATTCTGCT | 60°C |
| RPL7 | AATGGCGAGGATGGCAAG | TGACGAAGGCGAAGAAGC | 60°C |
| GAPDH | CAGTCAGCCGCATCTTCTTTTG | ACCAGAGTTAAAAGCAGCCCT | 60°C |
